# A systematic review of sustainable food systems identifies socio-economic pathways driving food systems transformations

**DOI:** 10.1038/s43016-026-01317-0

**Published:** 2026-03-16

**Authors:** Daniel Chrisendo, Sara Heikonen, Johannes Piipponen, Thomas Banafa, Delphine Deryng, Mohammad El Wali, Matias Heino, Xavier Irz, Mika Jalava, Josias Láng-Ritter, Rachel Mazac, Venla Niva, Mia Pihlajamäki, Marja Roitto, Hanna L. Tuomisto, Matti Kummu

**Affiliations:** 1 Water and Development Research Group, Aalto University, Espoo, Finland; 2 Department of Land Economy, University of Cambridge, Cambridge, UK; 3 Integrative Research Institute on Transformations of Human-Environment Systems (IRI THESys), Humboldt University of Berlin, Berlin, Germany; 4 European Center for Medium-Range Weather Forecasts (ECMWF), Bonn, Germany; 5 Department of Agricultural Sciences, University of Helsinki, Helsinki, Finland; 6 Department of Economics and Management, University of Helsinki, Helsinki, Finland; 7 GIScience for Sustainability Transformations, Aalto University, Espoo, Finland; 8 Stockholm Resilience Centre, Stockholm University, Stockholm, Sweden; 9 Helsinki Institute of Sustainability Science (HELSUS), University of Helsinki, Helsinki, Finland; 10 Natural Resources Institute Finland (Luke), Helsinki, Finland

**Keywords:** Sustainability, Socioeconomic scenarios, Agriculture

## Abstract

Socio-economic conditions influence the implementation of proposed solutions for transforming food systems. Here we systematically screen over 1,700 articles and select 349 for detailed review, investigating the role of socio-economic drivers in sustainable food systems transformations across different world contexts. We identify seven sustainable food systems transformations, including sustainable land resources and soil health, precision agricultural practices, diet change and novel food transition, good nutrition and health, food loss and waste reduction, healthy freshwater and marine ecosystems, and climate change mitigation and biodiversity conservation. We propose socio-economic pathways comprising specific socio-economic drivers needed for achieving them and provide actor-specific recommendations to support sustainable food systems transformations.

## Abstract (web summary)

Studies on food systems transformations are heterogeneous. This systematic review develops pathways aimed at six key food systems actors across socio-economic contexts that can shift food systems towards greater sustainability.

## Main

Scholars across disciplines have proposed potential solutions to transform food systems into ones that are socially, economically, environmentally and nutritionally sustainable^1^. Here food systems refer to the entire food supply chain, from farms (in agriculture, forestry and fisheries) to forks (at the consumer level, including the acquisition and consumption of food), encompassing a wide range of actors^2^. Sustainable food systems deliver food and nutrition security in ways that enhance economic profitability and social benefits, while ensuring positive or neutral environmental impacts^3^. Suggested solutions for sustainability include innovations to increase agricultural productivity (for example, Subramanian^4^), reduce carbon emissions (for example, Liu et al.^5^) and adopt more sustainable diets (for example, Treich^6^). However, implementing these solutions is challenging owing to the complex social and economic processes in which the food systems are embedded^7^. This raises the question of how socio-economic drivers support or hinder sustainable food systems transformations.

Some sustainable food systems solutions might be expensive, culturally unwelcome and politically challenging, which hinder their implementation^8^. For example, interventions to reduce food loss and waste are costly and require synergies across food supply chains^9^. While food waste reduction can improve food security in sub-Saharan Africa, it may lead to overconsumption in high-income regions due to falling food prices as more food becomes available on the market—a phenomenon known as a rebound effect^10^. Replacing meat with plant-based diets could reduce greenhouse gas emissions but has faced cultural and habit-driven reluctance^11^. It could also increase the risk of inadequate intake of nutrients such as protein, zinc, iron, calcium and vitamin B_12_ (refs. ^12,13^). In low-income countries, animal-source foods can help fight child stunting^14^. Therefore, global initiatives to reduce meat consumption should also promote nutrient-rich, plant-based foods to ensure that people get essential nutrients^12,13^. Meanwhile, agricultural technologies are not always adopted in regions where they are most needed^15^. Credit, insurance, subsidies and other market-related constraints are mentioned as the main barriers to adoption that policies could potentially overcome^16,17^.

Studies on sustainable food systems are heterogeneous, encompassing various disciplines, time scales, regions and topics with diverse results (for example, Béné^18^; Brouwer et al.^19^; Crippa et al.^20^; Popkin and Reardon^21^). This scattering makes it challenging to grasp key points, for example, for policymakers who rely on scientific findings to formulate effective, systems-relevant policies. Therefore, a clear and comprehensive overview of evidence for sustainable food systems transformations, especially from the perspective of socio-economic drivers, is needed but is missing from the existing literature. This paper helps close that gap through a systematic review of 349 articles published between 2015 and 2022 (see our study protocol in Chrisendo et al.^22^). We synthesize scientific knowledge on the role of socio-economic drivers in sustainable food systems transformations across geographical contexts (Methods). More specifically, we aim to (1) map food systems transformations identified in the literature and categorize them as farm side, fork side and farm to fork (that is, the linkage between production and consumption); (2) describe how socio-economic drivers help or hinder transformation in different regions; (3) develop pathways towards sustainable food systems; and (4) identify research gaps related to sustainable food systems transformations. We conclude by providing recommendations for six key actors to increase sustainability in food systems.

## Results

### Records of sustainable food systems literature

**Fig. 1:**
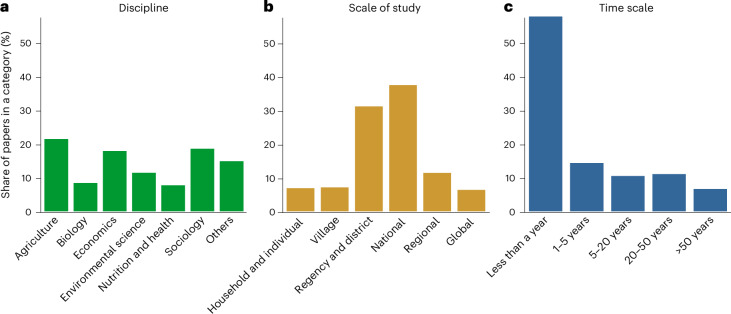
Descriptive summaries of articles included in this review. **a**–**c**, Share of publications in the most frequent categories across disciplines (**a**), the spatial scale (**b**) and the time scale (**c**). Note that a publication may include multiple categories of one variable, for example, multiple disciplines. Therefore, the sum of shares from all categories of a variable may be more than 100%.

The reviewed scientific articles report the effects of socio-economic drivers on food systems across all geographical regions. Studies investigating food systems in Europe and northern America make up 41% of the articles (Table 1). Around 15% of the articles are studies in eastern and southeastern Asia, and 10% are in sub-Saharan Africa. The rest of the world contributes less than 10% per region. Most articles come from agriculture, economics and sociology disciplines, with approximately 20% from each (Fig. 1a). Studies were mostly conducted at the national, regency and district levels, each with over 30% (Fig. 1b). Almost 60% of articles were of studies conducted within a 1-year period (Fig. 1c).

**Table 1:** Distribution of articles by geographical region

World regions	Number of articles	Share of database (%)
Global	32	9
Europe and northern America	143	41
Latin America and the Caribbean	28	8
Australia and Oceania	20	6
Eastern and southeastern Asia	51	15
Central and southern Asia	30	9
Northern Africa and western Asia	16	5
Sub-Saharan Africa	36	10

A publication may include multiple geographical regions; therefore, the sum of the shares of the database exceeds 100%.

### Food systems transformations and socio-economic drivers across regions and the supply chain

**Fig. 2:**
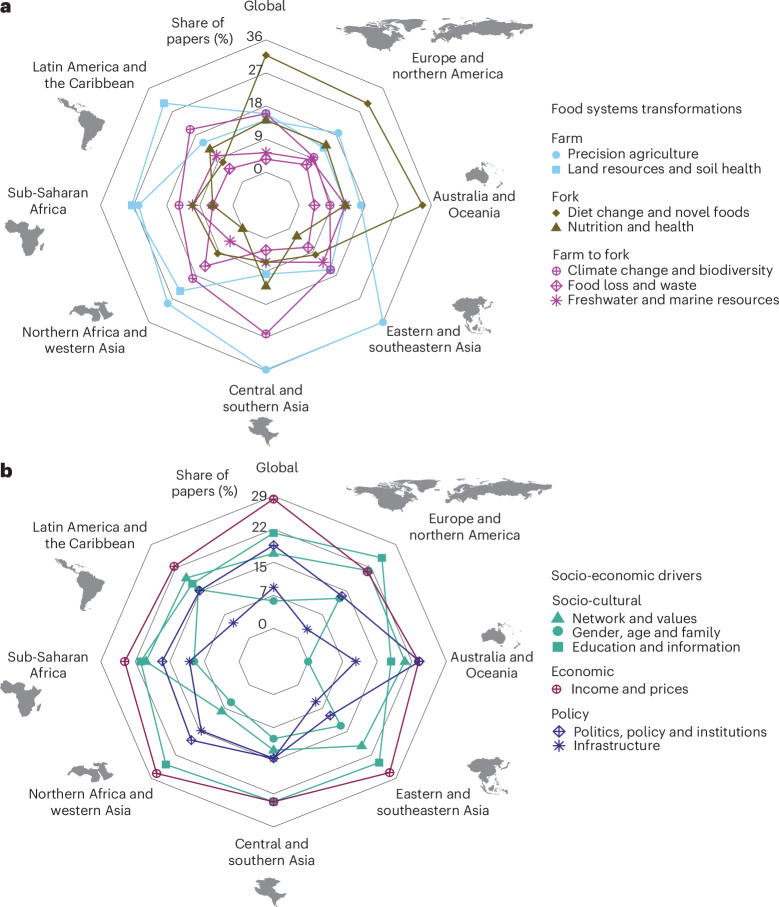
Food systems transformations and socio-economic drivers analysed in the reviewed articles by geographical region. **a**, Food systems transformations. **b**, Socio-economic drivers. Radar axes reflect the percentage of articles that mention each topic over the total number of articles for that region. ‘Global’ refers to global-scale studies, not the average or sum of all regions. Inset maps from Natural Earth (https://www.naturalearthdata.com).

We identified seven food systems transformations most frequently discussed in the literature (see Methods for selection criteria and definitions): on the farm side, (1) precision agriculture and (2) land resources and soil health; on the fork side, (3) diet change and novel foods and (4) nutrition and health; and topics overreaching from farm to fork, (5) climate change and biodiversity, (6) food loss and waste, and (7) freshwater and marine resources (Fig. 2a). The articles discuss socio-economic drivers that facilitate or hinder the transition to sustainable food systems. They are (1) network and values (for example, farmers’ organization, social media use, lifestyle); (2) gender, age and family (for example, producers and consumers’ gender and age, household size); (3) education and information (for example, years of schooling, information on health and environmental benefit of food); (4) income and prices; (5) politics, policy and institutions (including property rights); and (6) infrastructure (Fig. 2b).

Articles from different world regions focus on different types of food systems transformations. Studies at the global level, as well as in Europe and northern America, Australia and New Zealand, and Oceania, primarily focus on diet change and novel foods (Fig. 2a). Economic development and urbanization in Europe, North America, Australia and New Zealand have led to high meat consumption, raising questions about sustainability^23^. Meanwhile, Oceania has some of the highest rates of overweight and obesity among citizens^24^. Therefore, a relatively higher amount of research on diet change and consumption-side interventions in these regions was expected. With rising meat consumption in emerging economies and large-population countries such as Brazil, China and India^23^, the next hotspot of such research will probably be in those regions.

Most studies in Asia and northern Africa focus on precision agriculture (Fig. 2a). Many countries in these regions are middle-income nations experiencing rapid socio-economic changes. Dramatic increases in access to electricity enable the adoption of advanced technologies to improve crop yields and farm management^25^. However, the technology adoption rate is still lower than in high-income regions^26^, making research on these topics especially relevant. In sub-Saharan Africa and Latin America and the Caribbean, most studies focus on land resources and soil health (Fig. 2a). Sub-Saharan Africa has the lowest crop productivity^16^. Along with Latin America, it faces contentious land resource issues, frequently leading to disputes^27,28^, which can result in farmland loss, fewer agricultural job opportunities and, thus, lower food production^29^.

Looking at socio-economic drivers, the proportions of each driver covered in the reviewed literature are similar across all regions (Fig. 2b). Income and prices were the main socio-economic drivers discussed as affecting food systems. Reviewed articles show that producers’ adoption of and reluctance towards sustainable farming, as well as consumers’ preference for sustainable and healthy diets, are driven by economic considerations^30–34^. Education and information, and infrastructure and institutions, were also frequently discussed in the reviewed literature. Meanwhile, politics and policy were the least discussed drivers in sustainable food systems publications.

**Fig. 3:**
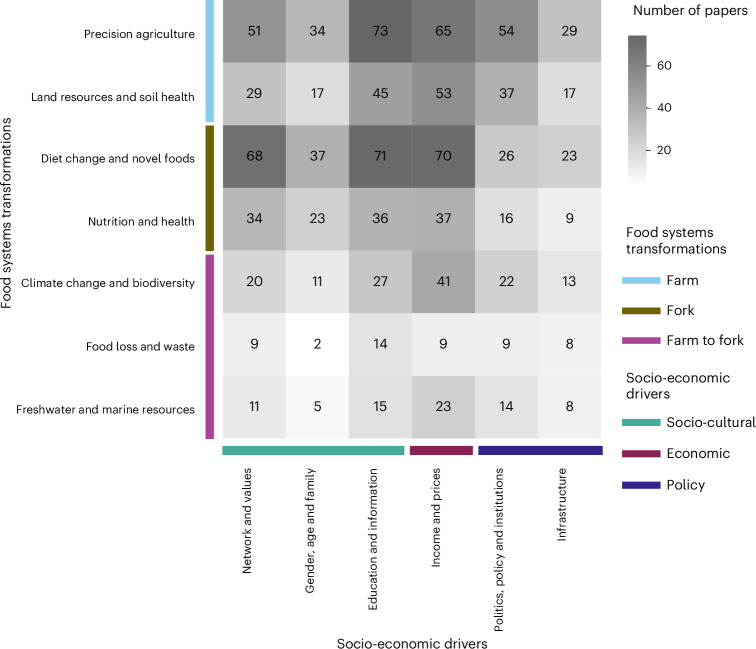
Frequency of socio-economic drivers discussed together with food systems transformations. The numbers and the background colour in the heat map grid show the number of papers that analysed each socio-economic driver together with each food systems transformation.

Studies on different food systems transformations discussed different socio-economic drivers (Fig. 3). For example, articles covering diet change and novel foods frequently discussed education and information (*n* = 71), income and prices (*n* = 70), and network and values (*n* = 68) as potential drivers. The literature shows that consumers with high incomes and education^30,35^, who receive information on the benefits of reducing meat consumption^36^ and who are surrounded by friends who eat plant-based diets^37^ tend to adopt more sustainable and healthy diets. Meanwhile, higher prices for novel or alternative foods may hinder adoption, especially among lower socio-economic groups^30,38^. However, Bunge et al.^39^ show that in higher-income settings, choosing whole-food or plant-based alternatives only slightly increases food expenditure while benefiting health and environmental outcomes. Articles on precision agriculture often examined education and information (*n* = 73), income and prices (*n* = 65), and politics, policy and institutions (*n* = 54). Research on other topics (nutrition and health, climate change and biodiversity, freshwater and marine resources, and land resources and soil health) also discussed income and prices. The most discussed socio-economic drivers in food loss and waste articles were education and information (*n* = 13).

### Pathways towards sustainable food systems

**Fig. 4:**
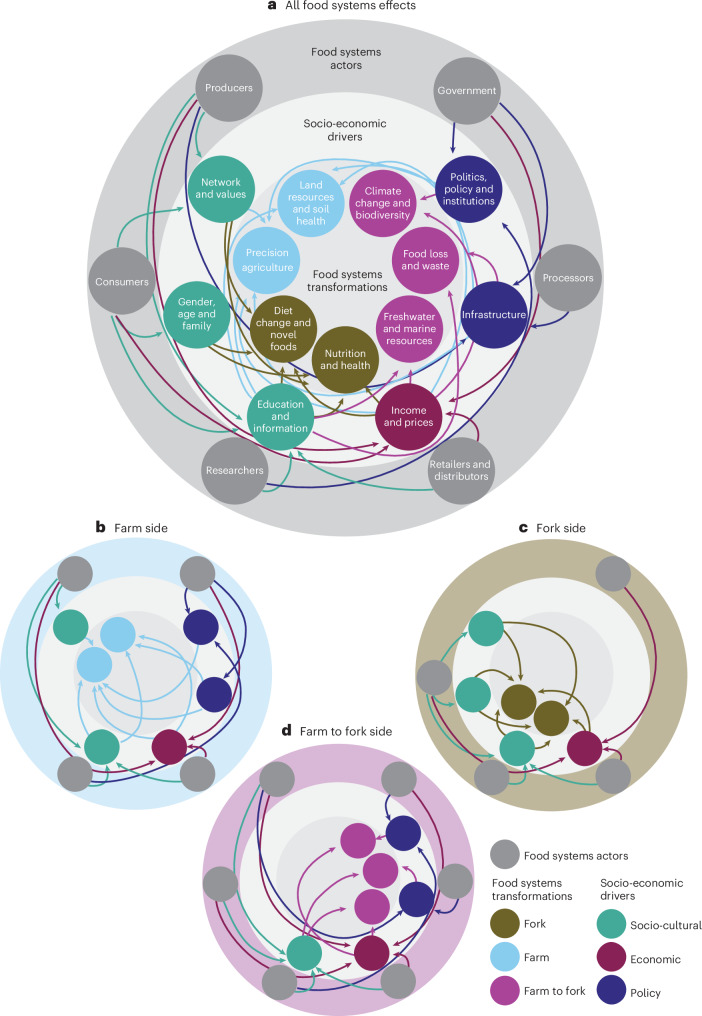
Interconnected relationalities among food systems transformations, socio-economic drivers and food systems actors. **a**, All food systems transformations. **b**, Farm side (land resources and soil health and precision agriculture). **c**, Fork side (diet change and novel foods and nutrition and health). **d**, Farm to fork side (climate change and biodiversity, food loss and waste, and freshwater and marine resources). The labels of circles in **a** also apply to **b**–**d** based on the exact position of the circles.

Studies have analysed topics related to food systems^8,19,40^. For example, Brouwer et al.^19^ reviewed food system frameworks, analyses and reports and their real-world relevance. Our study complements this by focusing specifically on socio-economic pathways. We show how food systems transformations and socio-economic drivers intertwine and, together, could promote sustainable food systems. Given the current literature, it is not possible to draw general conclusions on relative strengths, importance or dependencies among those variables. However, we provide targeted pathways for sustainable food systems at different food supply chain stages (Fig. 4a–d and Table 2), considering world regions and income levels. The pathways are based on our synthesis of the reviewed articles.

**Table 2:** Seven sustainable food systems transformations identified in the literature and respective socio-economic pathways

Sustainable food systems transformations	Socio-economic pathways to reach the transformations
(1) Sustainable land resources and soil health	Pathways for sustainable land use and soil health rely on farmer-driven knowledge sharing, community-led innovation and targeted training, particularly for older or less educated farmers. Networks, social media and trusted community figures support conservation agriculture and crop diversification. However, context-insensitive regulations and a lack of financial incentives can hinder progress. Policies based on scientific evidence, tailored to local needs and supported by land security and access to inputs are critical for transformation.
(2) Precision agriculture practices	Pathways for precision agriculture rely on farmer education, affordable technology, secure infrastructure and credit availability. Farmers adopt new technologies when motivated by profit, driven by yield increases or cost reductions. Yet, smallholders face economic and infrastructural barriers. Retailers play a vital role by facilitating markets for sustainably produced foods. Targeted financial support and user-friendly technologies tailored to local contexts will expand precision farming.
(3) Diet change and novel food transition	Transitioning to sustainable diets is influenced by cultural values, gender norms and generational attitudes. Women and younger people are more likely to adopt sustainable eating habits, while men and older people are less inclined, shaped by social identity and perceptions of masculinity. Strategies include using influencers, reshaping food norms and improving affordability and access to plant-based and novel foods. Policies such as subsidies for plant-based options, removing livestock subsidies and promoting sustainable meals through education and default placement can reshape consumer choices.
(4) Good nutrition and health	Improving nutrition and health involves aligning consumer knowledge, values and habits. Education and targeted communication through networks and digital platforms shift preferences towards healthier foods. Retailers influence purchasing through product placement, pricing and guidance. Ensuring healthy food is affordable and familiar—by teaching preparation and dispelling cost myths—enables healthier eating across socio-economic groups.
(5) Food loss and waste reduction	Reducing food loss and waste requires full supply chain interventions. In low-income regions, improving storage and transport infrastructure reduces farm-level losses, while addressing gender disparities in access to post-harvest resources enhances efficiency. In high-income regions, consumer behaviour is critical; waste declines when shopping is intentional and well planned. Public campaigns can tap into these behavioural drivers, while targeted support for women farmers and localized infrastructure upgrades can mitigate upstream losses.
(6) Healthy freshwater and marine ecosystems	Sustainable use of aquatic resources is shaped by economic incentives, consumer awareness and institutional support. Fishers respond to certification when backed by subsidies or policies, but high prices and low consumer awareness limit sustainable seafood uptake. Information campaigns, especially for men and older consumers, and better retail strategies can increase demand. Aligning policies and economic tools to favour sustainable fisheries is crucial for protecting aquatic ecosystems.
(7) Climate change mitigation and biodiversity conservation	Climate mitigation and biodiversity conservation require careful policy coordination and cross-sectoral collaboration. Unaligned policies can cause unintended environmental harms, stressing the need for evidence-based approaches. Interventions must balance between economic and environmental goals. Biodiversity-friendly practices are vulnerable to food safety rules or production incentives unless they are well coordinated. Financial instruments, flexible governance and stakeholder engagement are key to managing trade-offs and advancing sustainability.

#### Farm side

At the producer level, most of the reviewed research focused on land resources and soil health and precision agriculture (Fig. 4b). Subtopics included smart technologies^41^, conservation agriculture^42^, and crop rotation and diversification^43^. Information and knowledge about sustainable farming’s benefits and practicalities are often mentioned as important drivers for farmer adoption. Efficient knowledge transfer can occur through peer-to-peer learning in social networks (for example, farmer’s groups^44^) and social media (for example, farmer-to-farmer how-to videos in West Africa^45^ and farmer influencers in Hungary and the United Kingdom^46^). Carlisle^47^ shows that a community’s sense of belonging holds farmers accountable for growing organic food. These examples are found worldwide, in both higher- and lower-income regions. They highlight the importance of social capital, in which farmers trust peers and feel part of a community. Farmers find trying new practices or behaviours more rewarding when peers recommend them or have a positive attitude towards them. Therefore, disseminating knowledge and skills through organizations and social networks can efficiently promote sustainable practices.

Institutions and regulations sometimes act as barriers to sustainability for farmers in some regions. Elias and Marsh^48^ show that the cheap food policies in California, which favour urban consumers, force smallholder farmers to leave agriculture owing to low profits. Some sustainability-enhancing regulations are also reported as impractical, with farmers facing compliance barriers, for example, orange farmers in Egypt who struggle with global good agricultural practice standards^49^. In Europe, the legume sector—an important source of micronutrients and plant-based protein— receives less policy support than other staple crops^50^. Meanwhile, regulations not tailored to the regional context may hinder smallholder farmers from diversifying their crops or income sources^51,52^, and uncoordinated, unscientific legislation may not support sustainable outcomes. For example, China’s rice field protection policy contradicts wetland conservation efforts owing to unclear, poorly coordinated strategies^53^. Designing regulations that fit shifting stakeholder demands is challenging. Still, policy should be based on scientific evidence rather than political opinion, voter pressure or lobbying (for example, Sievert et al.^54^). Policies should also be flexible and adaptable to changing situations. For example, Australia’s food policy should better integrate Aboriginal peoples’ recommendations to account for their diets and cultures^55^.

Consistently, education was identified as a key driver for producers worldwide (Fig. 4b). Educated farmers were reported to use resources more efficiently and to adopt new practices more systematically^56–58^. However, older, less educated farmers tend to be slower to learn new methods^59,60^. It is more challenging for such a group to be aware of training opportunities, and they show limited acceptance of these issues owing to distrust of new farming methods and a lack of an innovation culture^61^. Therefore, special attention should be paid to targeting this type of farmer in any sustainable farming programme.

Producers’ decisions to adopt sustainable practices often relate to maximizing profit, saving money or a lack of funds (especially in lower-income countries), reflecting income and price as influential and commonly discussed factors across geographies and socio-economic drivers (Figs. 2b and 3). For example, Malawian farmers chose deep-bed farming—a conservation agriculture practice^42^—because it reduced production costs or increased yields. By contrast, high costs deter adoption, as seen among eastern Indian farmers who could not afford inputs, machinery or irrigation^62^. Increased access to credit, higher subsidies and incentives can make sustainable practices more viable; studies show that financial returns increase farmers’ willingness to adopt new technologies^63,64^. Concrete input packages, field demonstrations, farm visits and training can also increase adoption rates of improved technologies^61^. Secure infrastructure, such as land ownership, offers farmers the freedom to choose practices and plan long term. As an example, secure land ownership substantially increased the use of alternate wetting and drying irrigation among Bangladeshi farmers^65^. Retailers play an important role in influencing what and how farmers produce^66^. They can provide farmers with access to selling space for sustainable and healthy products^67,68^. This certainty motivates farmers to produce food more sustainably.

#### Fork side

Research on the fork side or consumer level was mainly conducted in high-income countries. At this stage, topics such as diet change and novel foods, and nutrition and health, dominate (Fig. 2a) and share similar socio-economic drivers (Fig. 4c). The first set of drivers—network and values—is contextual. They may or may not promote sustainable eating habits, such as choosing healthy, local, organic or plant-based foods and reducing meat consumption. For example, people who adopt either the Mediterranean diet (seen as more sustainable) or the meat-rich diet (less sustainable) mention both culture and heritage as reasons for following these diets^35,54,69^. Therefore, redefining values and norms that consider environmental protection and animal welfare may foster the construction of sustainable eating motives. Yet, the complexity of these factors challenges the prospect and speed of change.

Reviewed articles show that women tend to eat more sustainably and healthily than men. Women consume less meat and more fruits and vegetables, and are more open to novel and alternative foods^36,70^. This behaviour links to traditional feminine and masculine identities, especially in high-income countries^54^—of note, the literature entirely neglects non-binary gender inclusivity. In these regions, meat is associated with protein, power, strength, muscle building, masculinity and being a ‘real’ man^54,71^. Younger generations also typically have more positive attitudes towards diet change and novel foods^31,72^. However, there is no consensus on how to classify younger and older ages (children are excluded). For example, Annunziata et al.^35^ define younger people as under 64 years old, who are more likely to consume local and organic food. Meanwhile, Mancini et al.^73^ define people aged 60 as older, who consume less sustainably. Accordingly, programmes to increase the adoption of sustainable diets might have a greater impact when effectively targeting men and older consumers. For example, some movements, activism and influencers contest the concept of hegemonic masculinity around physical strength and muscle. They instead promote ‘meatless masculinity’, arguing that men who do not eat meat are masculine too^74–76^.

Consumers can shift their attitudes towards sustainability by gaining more education and information about sustainable food choices, including health and environmental benefits^32,70^. Similar to producers adopting sustainable farming practices, the articles consistently show that effective ways to disseminate information are through more personal social networks and social media promoted by friends, family, influencers and celebrity chefs, rather than through broader, less personal campaigns^77,78^. Setting sustainable options as the default meal in restaurants or catering establishments and supplementing them with nutritional labels on the menu increase sustainable food consumption^79^. At home, consumers’ knowledge of food preparation, especially with novel and alternative foods, helps adoption^72,80^. The perceived high price of plant-based meat substitutes and other alternative foods hinders consumers from choosing these food options^70^. However, in particular contexts, the high cost is really only a perception rather than a reality (for example, Bunge et al.^39^). A study in Mexico shows that people can adopt sustainable, healthy diets without extra cost^81^. Subsidizing plant-based meat substitutes and removing subsidies for livestock and meat production make novel and alternative foods more competitive and boost consumption^54,72^.

Consumers also acquire information about sustainable and healthy foods from retailers^82^. Retailers can influence consumer choices through prices, product layout and placement, on-shelf availability, and in-person assistance and consultation^83–85^. Niche retailers such as farmers’ markets differentiate themselves from mainstream retailers by offering high-quality products (fresh, local, seasonal, organic, environmentally friendly) and by reconnecting producers and consumers through short supply chains and community building^86–88^.

#### Farm to fork side

Research on food loss and waste, climate change and biodiversity, and freshwater and marine resources spans all stages of the food supply chain (Fig. 4d). Food loss could be reduced by improving infrastructure, such as storage and transportation facilities, especially in low-income countries^63^. Gender-responsive strategies are needed to address higher post-harvest losses among women in sub-Saharan Africa due to differences in facility access (for example, Kikulwe et al.^89^). At the consumer level, studies link food waste to shopping habits in high-income regions. Food waste is lower when consumers create a purchase plan or shopping list, check their inventory before shopping or shop more frequently rather than buying large amounts of food infrequently^90,91^. These insights can be used when designing campaigns and interventions to target consumers’ awareness of food waste reduction.

Various policies were linked both positively and negatively to climate change and biodiversity. Tanentzap et al.^92^ argue that government price-support policies, such as minimum commodity prices or import tariffs, are reported to have reverse implications on climate change and biodiversity due to overproduction. Elias and Marsh^48^ provide another example, showing that California’s food safety regulations require farmers to create more sterile farm environments, which decreases farm biodiversity. These examples highlight that trade-offs often occur among social, economic and environmental objectives when a policy is proposed, underscoring the importance of collaboration for successful policy implementation and goal achievement^93^.

Sustainable seafood production and consumption were affected by socio-economic drivers similar to those in terrestrial agriculture. Fishers’ participation in sustainable certification schemes is motivated by economic incentives and government support^94^. However, higher prices and a lack of information about the health and environmental benefits of sustainable seafood are barriers to its consumption^80^. This again highlights the critical role of retailers^84^. Providing financial aid, such as subsidies for fisheries and sustainable seafood products, could incentivize sustainable production and consumption practices. Information dissemination—especially targeting men and older consumers, who are less likely to consume sustainable seafood^80^—will also be beneficial.

On the basis of our analysis and the desired food systems transformations discussed in the literature, we propose socio-economic pathways based on socio-economic drivers for achieving seven sustainable food systems transformations (Table 2). To achieve these transformations, several common strategies and challenges emerge. Central components include education, training and community engagement, recognizing the influence of demographics such as gender, generation and socio-economic status. Economic incentives, infrastructure development and targeted policies are crucial for adoption and behaviour change. Integrating local contexts into policy design and fostering collaborations across sectors are indispensable in transforming agriculture and food systems towards sustainability.

## Discussion

### The need for sustainable food systems principles and effects measurements

Sustainable food systems are a broad topic; it is foreseeable that our initial screening identified documentation with varied foci, methods and geographical scopes. Different regions have different priorities. In lower-income regions, research often looks at limited credit, a lack of agricultural inputs and poor infrastructure as barriers to food security and to enhancing farmer welfare (for example, Mvula and Dixon^42^; Urfels et al.^62^). In high-income countries, where food access is secure and producers and consumers are wealthier, topics of environment, animal welfare and food quality are more common (for example, Sans and Combris^23^; Sievert et al.^54^). Despite myriad documentation, we identified two main research gaps: the need for clear sustainable food systems principles and quantitative measurement of sustainability effects.

Many researchers use the term ‘sustainable food systems’, but often without clear principles. We found many articles that mention it as a buzzword and do not analyse or define it, so we had to exclude them. The phrase was also attributed to multiple meanings by different authors, making summaries difficult to produce. Our findings align with those of Béné et al.^8^, who found that the concept of sustainability in food systems is still poorly understood, applied in varied ways and often interpreted too narrowly, which leads to conflicting solutions and priorities. They also found that social and economic dimensions receive little attention in sustainable food systems research. Another study found that environmental, social, economic and nutritional dimensions of sustainability often compete and are hard to balance^95^. Collaboration and inclusion among researchers are vital to shaping these definitions and policies and to promoting sustainable food systems.

Some articles propose frameworks for greater cross-sector collaborations, public–private partnerships and knowledge sharing^96,97^, which could clarify the intrinsic principles necessary to track progress towards sustainable transformations and achieve desired outcomes^98^. Such collaborations are necessary to address the global biodiversity and climate crises^99^, to allow effective management of natural resources^93^ and to resolve the data scarcity problem^100^. Yet, some recommendations are often too general and lack critical or nuanced steps to achieve them. For instance, Zaharia et al.^83^ call for a collaboration platform for European Union countries. However, measures for implementing it successfully need to be researched.

Throughout our review, we observed a paucity of studies that quantify the effects of the proposed socio-economic drivers or certain sustainability-enhancing proposals. For example, one study claims that community gardens in Edinburgh benefited health and the environment^101^, and another study focuses on how novel food products could address sustainability^102^. However, in those studies, the benefits are only assumed rather than measured. We saw similar gaps in other studies^41,55,83^.

Many empirical studies are of limited relevance, often consisting of short or small-scale case studies. For example, one study claims that a 4 h week^−1^ Canberra farmers’ market advances an equitable food system^87^. Such a study plays an essential role in describing local nuances, but the scalability needs to be investigated in future research. Some papers dealt only with a single driver. For example, a study in Turkey argues that sustainable food consumption can be achieved by increasing consumer awareness^103^, and a study in Poland shows that the adoption of biochar could be increased by providing producers with more information^104^. However, other socio-economic drivers, such as values or prices that may play a role, should have been considered for feasibility in both studies. Therefore, their relevance to the broader food systems is not always clear. Many proposals do not consider costs and benefits, leading to unrealistic recommendations (for example, Wezel et al.^105^).

### Concluding remarks

We reviewed articles from 2015 to 2022 on how socio-economic drivers transform sustainable food systems. We present socio-economic pathways for key transformations in the literature, namely (1) sustainable land resources and soil health, (2) precision agricultural practices, (3) diet change and novel food transition, (4) good nutrition and health, (5) food loss and waste reduction, (6) healthy freshwater and marine ecosystems, and (7) climate change mitigation and biodiversity conservation (Table 2). The articles consistently identify similar drivers across regions, revealing outcomes of sustainable food systems, such as environmentally friendly practices and healthy, accessible food for all. However, the focus of research often differs by regions’ income level. In lower-income regions, studies focus on producers, land resources and productivity, while in higher-income regions, they focus on consumers, diet change, novel foods and nutrition. Researchers also prioritize sustainability’s social, economic, environmental and nutritional aspects differently worldwide, often leading to trade-offs among them.

Our review led us to recommend key actions for each of six food systems actor groups: (1) producers, (2) processors, (3) retailers and distributors, (4) consumers, (5) researchers and (6) governments (Box 1). Each group has unique roles, challenges, capacities and incentives for advancing sustainability, so recommendations must account for the complexity of food systems. Implementing these actions can promote seven sustainable food systems transformations in varied contexts. Finally, we urge researchers and policymakers to rigorously measure the effects of socio-economic drivers, use large-scale ex ante modelling and monitor policy initiatives. Without these efforts, scattered food systems research remains too conceptual for strong policy guidance. Collaboration among actors is crucial to setting clear goals and developing policies that foster commitment to sustainable food systems.

Box 1 Actor-specific recommendations to support the seven sustainable food systems transformations

**Table:** 

	Strong social capital and agricultural support for producers	The main factors hindering producers from practising sustainable farming are limited knowledge of novel agricultural innovations, insufficient technical skills and high costs. Especially for those whose access is limited (for example, small-scale, elderly, women and low-educated farmers), social capital and agricultural support could be highly beneficial. Trust among farmers is an important factor in increasing the adoption of sustainable farming practices, as farmers tend to model each other. Trust can be established through cooperatives or other farmers’ groups, in which information, training, subsidy, incentives, technology and input packages could be shared.
	Food innovation and transparent labelling by processors	Processors should invest in sourcing ingredients from farmers who practise sustainable farming. They can innovate and promote healthy, affordable and culturally acceptable plant-based products. Adopting transparent sustainability labelling and certification can help meet the growing consumer demand for sustainable and healthy food while influencing producers to adopt more sustainable practices.
	Better infrastructure and in-store strategy for retailers and distributors	The availability of newly developed storage and distribution infrastructures is needed to reduce food loss and waste, especially for highly perishable products (for example, fruits and vegetables) in lower-income countries. Retailers can incentivize sustainable farming by ensuring timely and equitable access to sustainable inputs needed by producers and offering market access and shelf space for sustainably produced foods. On the consumer end, retailers should promote sustainable and healthy food choices through strategic pricing, product placement, clear sustainability labelling and in-store education.
	Provision of information and lower consumer prices for healthy, sustainable options	Information on the health and environmental benefits of sustainable diets should be disseminated clearly and widely, especially to men and elderly consumers and citizens who are more reluctant to reduce meat consumption. Change to a more sustainable diet could be enforced through a lower price, for example, through subsidy, especially on meat alternatives and organic products, or by higher disincentive taxes on impactful food items.
	Accessible findings and innovative research by researchers	Scientific findings regarding food systems should be accessible to the broader public as necessary information in making food-related decisions. There is a need to conduct research at different scopes and scales to validate the applicability of the proposed solutions. Measures of the effects of various proposals on sustainability and their implementation costs should be rigorously quantified to provide realistic policy recommendations, and should be communicated clearly and accurately to targeted audiences for different use cases. Much existing research assumes positive effects of investigated measures without testing the hypotheses.
	Investment and collaborative policy-making by governments	Policymakers, particularly governments, should create policies and finance programs tailored to facilitate sustainable food systems transformations in their respective socio-political contexts. Such context-specific instruments should be based on the three dimensions of sustainability and include objectives and measures for each stage of the food supply chain. In addition, cross-sectional collaborations and public–private partnerships that foster commitment towards sustainable food systems should be enforced and incentivized.

Credit: icons parts adapted from SVG Repo under CC0.

## Methods

**Extended Data Fig. 1:**
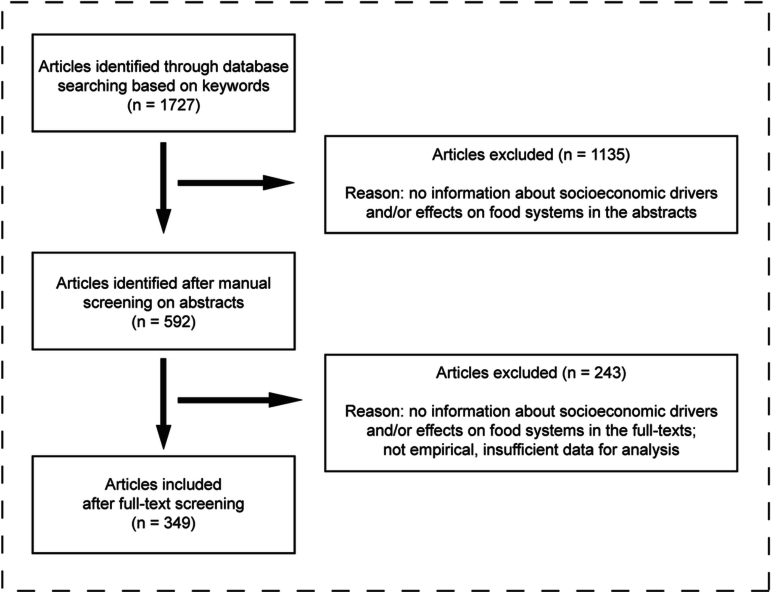
**PRISMA diagram for the article selection flow**.

We systematically reviewed the literature in accordance with the Preferred Reporting Items for Systematic Reviews and Meta-Analysis (PRISMA) guidelines, which map the flow of information throughout the article search (Extended Data Fig. 1).

### Protocol and registration

We registered the detailed screening protocol for this article on Nature Protocol Exchange. The protocol was published on 22 March 2022^22^.

### Database search

We used search strings that represent two combined concepts: food systems (‘food systems’ OR ‘agrifood systems’ OR ‘agriculture’ OR ‘seafood’ OR ‘livestock’ OR ‘food distribution’ OR ‘diet’ OR ‘food consumption’) AND socioeconomic drivers (‘socioeconomic’ OR ‘social’ OR ‘economic’). To the food systems concept, we added prefixes to capture sustainability terms, including transition towards sustainable food system and its feasibility (‘sustainable’ OR ‘transition’ OR ‘feasible’). We considered various spellings and grammatical numbers (that is, singular or plural). We conducted the search in the Scopus database on the titles, abstracts and keywords of articles. From the initial screening, we retrieved a total of 1,727 articles.

### Inclusion and exclusion criteria

We conducted training with key details for our coders (*n* = 12) as a guide to perform the abstract and full-text reading, eligibility and quality assessment, and data extraction. We focused on articles that draw connections between food systems and sustainability. To be included, articles must analyse food systems solutions and opportunities and, at the same time, the socio-economic drivers relevant to improving food systems or tackling food system challenges. If one or both were missing, or if studies deviated from the intended research scope or lacked sufficient detail, the literature was excluded. We included only empirical articles, modelling studies based on empirical data (primary and secondary) and reviews of empirical articles. We excluded concept and theoretical papers not grounded in empirical research, simulations not based on empirical data, reports, books, non-peer-reviewed scientific papers (that is, working and conference papers), comments and other grey literature. The inclusion and exclusion criteria (Extended Data Table 1) are converted to a set of decision steps:

1. Do the articles have anything to do with sustainable food systems? If the articles do not draw connections with sustainable food systems, then the answer is no, and we excluded the articles. If the answer is yes, we proceeded to criterion 2.
2. Do the articles discuss solutions or opportunities to transform food systems or tackle food system challenges? If the articles do not discuss food solutions and opportunities, then the answer is no, and we exclude these articles. If the answer is yes, we proceed to criterion 3.
3. Do the articles analyse socio-economic drivers relevant to transforming food systems or tackling food system challenges? If the articles do not discuss socio-economic attributes, then the answer is no, and we excluded the articles. If the answer is yes, we proceeded to criterion 4.
4. Do the articles report on the analysis of empirical data, modelling studies based on empirical data (primary and secondary) or a systematic review of empirical research? If the articles present concepts and theories that are not grounded in empirical research or results of simulations that are not based on empirical data, then the answer is no. If the answer is yes, we included the articles in the sample.

Sustainable food systems are one central point of the sustainable development goals set in 2015. To reflect on publications after the set-up of the sustainable development goals and to assess socio-economic drivers that are more relevant to current food systems, we reviewed literature published in 2015 until the publication of the screening protocol (22 March 2022). We included only articles published in English and considered literature of various disciplines, geographical ranges and time scales. The selection of articles should also be sufficiently broad and not dominated by a single author, laboratory or institution.

We assessed whether the articles met the inclusion criteria based on their abstracts and included 592 articles for full-text reading. Afterwards, we again executed the assessment of inclusion criteria in the full text. Eventually, 349 articles had sufficient information for data extraction for our analysis (Extended Data Fig. 1). The author with the most articles has only 6 articles (1.7%) included, and together, the top 10 authors account for 12%. This concludes that a handful of authors do not dominate the articles we selected.

### Coding, data extraction and data cleaning

The abstract and full text were read by two different coders. We coded and extracted information on food systems transformations, socio-economic drivers, disciplines, geographical range, scale of study and time scale (Extended Data Table 2). Our team conducted the extraction as much as possible based on their expertise to ensure quality and minimize bias. For example, food loss and waste experts reviewed the literature on those topics. Seafood literature was reviewed by researchers who have studied marine science and policy. When a researcher was unsure how to characterize the information conveyed in the articles, one or more additional researchers were also assigned to read the full text and code the information. We developed a table in Microsoft Excel to store the data, allowing our researchers to cross-check, give feedback and validate each other’s work. Following the extraction, a small team of researchers (*n* = 2) cleaned the data to ensure that the codes were consistent before performing the main analysis.

### Selection, definition and categorization of food systems transformations and socio-economic driver categories

We have selected categories as granular as possible for the variables of food systems transformations and socio-economic drivers (Extended Data Table 2), critically extracted from the scientific literature. Initially, the list contained more categories (see protocol in Chrisendo et al.^22^). As we reviewed the articles and identified additional relevant categories, some categories were added to the list, thereby minimizing the chance of overlooking important categories. For brevity and comprehensibility, we finally grouped the categories based on similarities and logical justifications of how the categories are analysed in the articles.

The definitions and groupings of some categories are more apparent than others. We assigned the categories of food systems transformation to farm, fork and farm to fork sides based on how they were analysed in the majority of the screened articles. Land use and soil health and precision agriculture are related to farming practices and analysed on the producer side (farm side). Articles about diet change and novel foods and nutrition and health often discuss the willingness of consumers to adopt sustainable, novel and nutritious diets and food (fork). These categories are closely linked and sometimes overlap. However, as changes to, for example, plant-based and novel food diets are not necessarily nutritious and healthy, we separated these categories. Meanwhile, articles about climate change and biodiversity, food loss and waste (that is, food that is not consumed), and freshwater and marine resources (that is, aquatic resources) cover topics on the producer and consumer side (farm to fork).

For socio-economic drivers, we categorized network and values; gender, age and family; and education and information as socio-cultural drivers. Income (that is, the initial financial situation of food system actors to adopt sustainable practices) and prices (that is, the prices that consumers pay to adopt sustainable and healthy diets, producers pay to adopt sustainable farming practices and producers receive for selling crops) are part of the economic drivers. Infrastructure (for example, road and market access) and politics, policy and institutions (for example, formal and informal rules) are categorized as policy drivers.

### Analysis and synthesis of results

#### Descriptive analysis

Basic descriptive statistics were conducted to show the total number and share of literature based on four variables: geographical range, discipline, scale of study and time scale (Table 1 and Fig. 1). For these variables, one paper could cover multiple categories (for example, multiple disciplines); therefore, the sum of the share of articles in categories of a variable might be more than 100%. For disciplines, we showed the six most frequent disciplines individually and combined the remaining disciplines under ‘Others’. Furthermore, we visualized the share of articles that discuss food systems transformations and socio-economic drivers by world region (Fig. 2), as well as the frequency of socio-economic drivers discussed together with each food systems transformation (Fig. 3).

#### Socio-economic pathways towards sustainable food systems

After reading the articles and extracting the data, our full-text reviewers provided their reflections on the literature, including socio-economic drivers that they found important (that is, most commonly mentioned or most influential based on results in the articles) for sustainable food systems and various food system actors, the current research gaps and recommendations to move forward. A small team of researchers (*n* = 5) synthesized these reflections and drew interconnections among food system actors, socio-economic drivers and food systems transformations to provide targeted pathways and recommendations for establishing sustainable food systems for each sector of the food supply chain, considering world regions and income levels, where relevant. This synthesis was used to develop the interconnections among food systems transformations, socio-economic drivers and food system actors in Fig. 4, the socio-economic pathways towards different goals of sustainable food systems in Table 2, the actor-specific recommendations for sustainable food systems transformations in Box 1 and the identification of research gaps.

### Deviations from protocol

During the review process, two minor deviations from the protocol occurred during data extraction and coding. However, they did not affect the soundness of this research. First, due to data availability and to simplify the presentation of results, we removed, added and merged some categories in some variables (see our study protocol in Chrisendo et al.^22^ for the original categories). For the second deviation, we generated a new variable, time scales, consisting of different categories ranging from less than a year to more than 50 years.

### Reporting summary

Further information on research design is available in the Nature Portfolio Reporting Summary linked to this article.

## Supplementary information

Reporting Summary

## Figures and Tables

**Extended Data Table 1:**
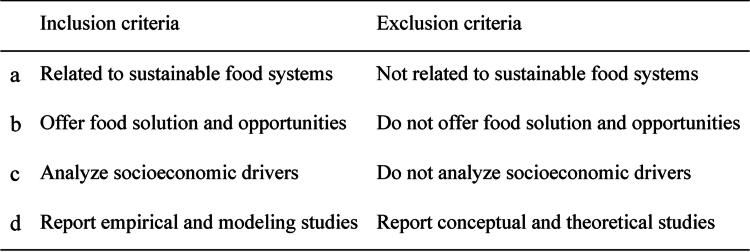
The inclusion and exclusion criteria of reviewed articles

**Extended Data Table 2:**
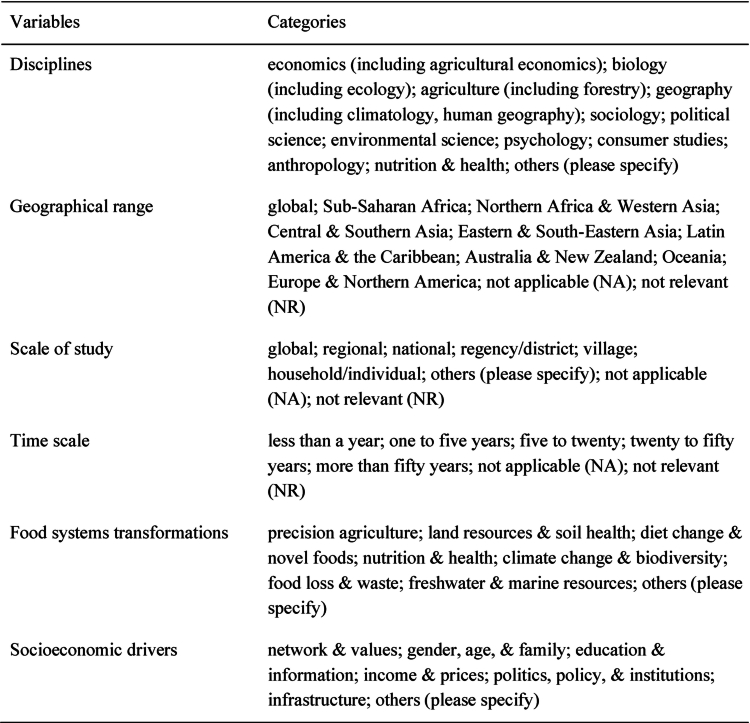
Categories of major variables

## Data Availability

Data from this study, including a list of papers reviewed, are available via Zenodo at 10.5281/zenodo.18015221 (ref. ^106^).

## Code availability

The code for creating visualizations of descriptive statistics is available via Zenodo at 10.5281/zenodo.18015221 (ref. ^106^).
