# Supplementary material for: A systematic review of sustainable food systems identifies socio-economic pathways driving food systems transformations
Source: Nat Food. 2026 Mar 16;7(3):234–46. doi: 10.1038/s43016-026-01317-0 (PMC13021506; doi:10.1038/s43016-026-01317-0)
Supplement: Supplementary file 1 — Reporting Summary [file 43016_2026_1317_MOESM1_ESM.pdf]

nature portfolio

Corresponding author(s): Daniel Chrisendo; Matti Kummu

Last updated by author(s): Jan 12, 2026

# Reporting Summary

Nature Portfolio wishes to improve the reproducibility of the work that we publish. This form provides structure for consistency and transparency in reporting. For further information on Nature Portfolio policies, see our Editorial Policies and the Editorial Policy Checklist.

## Statistics

For all statistical analyses, confirm that the following items are present in the figure legend, table legend, main text, or Methods section.

| n/a | Confirmed | |
|---|---|---|
| ☒ | ☐ | The exact sample size (*n*) for each experimental group/condition, given as a discrete number and unit of measurement |
| ☒ | ☐ | A statement on whether measurements were taken from distinct samples or whether the same sample was measured repeatedly |
| ☒ | ☐ | The statistical test(s) used AND whether they are one- or two-sided<br>*Only common tests should be described solely by name; describe more complex techniques in the Methods section.* |
| ☒ | ☐ | A description of all covariates tested |
| ☒ | ☐ | A description of any assumptions or corrections, such as tests of normality and adjustment for multiple comparisons |
| ☒ | ☐ | A full description of the statistical parameters including central tendency (e.g. means) or other basic estimates (e.g. regression coefficient) AND variation (e.g. standard deviation) or associated estimates of uncertainty (e.g. confidence intervals) |
| ☒ | ☐ | For null hypothesis testing, the test statistic (e.g. $F$, $t$, $r$) with confidence intervals, effect sizes, degrees of freedom and $P$ value noted<br>*Give P values as exact values whenever suitable.* |
| ☒ | ☐ | For Bayesian analysis, information on the choice of priors and Markov chain Monte Carlo settings |
| ☒ | ☐ | For hierarchical and complex designs, identification of the appropriate level for tests and full reporting of outcomes |
| ☒ | ☐ | Estimates of effect sizes (e.g. Cohen's *d*, Pearson's *r*), indicating how they were calculated |

*Our web collection on statistics for biologists contains articles on many of the points above.*

## Software and code

Policy information about availability of computer code

| | |
|---|---|
| Data collection | We used the Scopus database to collect the research articles, and no software was used to collect data from the articles. |
| Data analysis | We used R version 4.3.2 to produce figures 1, 2, and 3 in the manuscript. The analysis code is available at https://doi.org/10.5281/zenodo.18015221 |

For manuscripts utilizing custom algorithms or software that are central to the research but not yet described in published literature, software must be made available to editors and reviewers. We strongly encourage code deposition in a community repository (e.g. GitHub). See the Nature Portfolio guidelines for submitting code & software for further information.

## Data

Policy information about availability of data

All manuscripts must include a data availability statement. This statement should provide the following information, where applicable:

- Accession codes, unique identifiers, or web links for publicly available datasets
- A description of any restrictions on data availability
- For clinical datasets or third party data, please ensure that the statement adheres to our policy

Data from this study, including a list of articles reviewed are openly available at https://doi.org/10.5281/zenodo.18015221. The code used for creating the figures is openly available at https://doi.org/10.5281/zenodo.18015221.

## Human research participants

Policy information about studies involving human research participants and Sex and Gender in Research.

| | |
|---|---|
| Reporting on sex and gender | We did not have human research participants |
| Population characteristics | We did not have human research participants |
| Recruitment | We did not have human research participants |
| Ethics oversight | We did not have human research participants |

Note that full information on the approval of the study protocol must also be provided in the manuscript.

# Field-specific reporting

Please select the one below that is the best fit for your research. If you are not sure, read the appropriate sections before making your selection.

☐ Life sciences ☒ Behavioural & social sciences ☐ Ecological, evolutionary & environmental sciences

For a reference copy of the document with all sections, see nature.com/documents/nr-reporting-summary-flat.pdf

# Behavioural & social sciences study design

All studies must disclose on these points even when the disclosure is negative.

| | |
|---|---|
| Study description | The study is a systematic review with quantitative and qualitative data (mixed-method). |
| Research sample | The research sample consists of articles that analyze the socioeconomic drivers of sustainable food systems worldwide. The sample chosen allows us to provide global analysis. |
| Sampling strategy | We conducted keyword-based searches on the titles and keywords sections in the Scopus database, followed by abstract and full-text readings. For more detailed please see the protocol: https://dx.doi.org/10.21203/rs.3.pex-1863/v1 |
| Data collection | The data was collected through a full-text reading of the research articles and reported in a Microsoft Excel sheet. |
| Timing | We collected research articless published in and after 2015, the year the Sustainable Development Goals were set up, until March 2022, when we started the keyword-based searches. The data collection through Abstract and full-text readings was conducted between April 2022 and the end of 2023. |
| Data exclusions | Research articles were excluded when they were not related to the sustainable food system topic, did not discuss food solutions/ opportunities, did not discuss socioeconomic drivers, and were not empirical or modeling studies. |
| Non-participation | Not applicable. |
| Randomization | Not applicable. |

# Reporting for specific materials, systems and methods

We require information from authors about some types of materials, experimental systems and methods used in many studies. Here, indicate whether each material, system or method listed is relevant to your study. If you are not sure if a list item applies to your research, read the appropriate section before selecting a response.

## Materials & experimental systems

| n/a | Involved in the study |
| --- | --- |
| ☒ | ☐ Antibodies |
| ☒ | ☐ Eukaryotic cell lines |
| ☒ | ☐ Palaeontology and archaeology |
| ☒ | ☐ Animals and other organisms |
| ☒ | ☐ Clinical data |
| ☒ | ☐ Dual use research of concern |

## Methods

| n/a | Involved in the study |
| --- | --- |
| ☒ | ☐ ChIP-seq |
| ☒ | ☐ Flow cytometry |
| ☒ | ☐ MRI-based neuroimaging |
